# Interplay between brain-specific microRNAs and Alzheimer’s disease

**DOI:** 10.4103/NRR.NRR-D-25-00190

**Published:** 2025-07-05

**Authors:** Nathan Tinu, Bhupender Sharma, Daniela Rodarte, Rajkumar Lakshmanaswamy, Subodh Kumar

**Affiliations:** 1 Center of Emphasis in Neuroscience, Department of Molecular and Translational Medicine, Paul L. Foster School of Medicine, Texas Tech University Health Sciences Center El Paso, TX, USA; 2 L. Frederick Francis Graduate School of Biomedical Sciences, Texas Tech University Health Sciences Center El Paso, TX, USA; 3 Center of Emphasis in Cancer, Department of Molecular and Translational Medicine, Paul L. Foster School of Medicine, Texas Tech University Health Sciences Center El Paso, TX, USA

**Keywords:** Alzheimer’s disease, biomarker, brain function, microRNAs, therapeutics

## Abstract

Alzheimer’s disease is a progressive neurodegenerative disease characterized by memory decline and the accumulation of abnormal protein aggregates in the brain. While the precise cause of Alzheimer’s disease remains under investigation, recent research suggests that dysregulation of brain-specific microRNAs (miRs) plays a significant role in Alzheimer’s disease pathogenesis. Brain-specific miRs are predominantly expressed within the central nervous system and are crucial for neuronal development, and function, potentially in brain disorders. This review identifies some key brain-specific miRs in Alzheimer’s disease, including miR-9, miR-26b, miR-34a, miR-107, miR-124, miR-125b, miR-128, miR-132, miR-146a, miR-155, miR-219, miR-501-3p, and miR-502-3p. The review also shed light on the brain-specific location of these miRs, their dysregulation in Alzheimer’s disease, and how they are involved in disease progression. Apparently, these brain-specific miRs modulate specific genes and are therefore crucial for various cellular processes, including autophagy, cell cycle, tau phosphorylation, amyloid-beta production, and neuroinflammation. Moreover, these miRs are potent disease-modifying factors and their expression levels could serve as potential biomarkers for diagnosing or monitoring Alzheimer’s disease progression.

## Introduction

Alzheimer’s disease (AD) is a progressive and irreversible neurodegenerative disorder, representing the most common cause of dementia affecting millions of individuals globally. It is characterized by a gradual decline in cognitive function, primarily affecting memory, but also encompassing other critical domains such as language, reasoning, judgment, and executive function (Kshirsagar et al., 2022). The neuropathological hallmarks of AD are the accumulation of abnormal protein aggregates within the brain parenchyma. These include amyloid plaques, primarily composed of amyloid-beta (Aβ) peptides, and neurofibrillary tangles (NFTs), composed of hyperphosphorylated tau protein (Reddy et al., 2017). The Aβ peptide is a naturally occurring fragment derived from the amyloid precursor protein (APP) through sequential cleavage by beta- and gamma-secretase enzymes. However, in AD, Aβ aggregates fail to be cleared efficiently, leading to their accumulation and plaque formation (Hur, 2022). NFTs, conversely, arise from the abnormal hyperphosphorylation of tau protein, a crucial component of the microtubule network within neurons. These hyperphosphorylated tau proteins lose their functional capacity and aggregate into tangles, disrupting neuronal structure and function (Brion et al., 2001). The precise chain of events leading to AD pathogenesis remains an active area of research. However, the accumulation of Aβ plaques and NFTs is believed to be central to a neurotoxic cascade (Guo et al., 2020). Aβ plaques are thought to disrupt synaptic function, and communication between neurons, and trigger inflammatory responses within the brain (Kumar and Reddy, 2020). NFTs contribute to neuronal dysfunction by impairing axonal transport, the vital process by which neurons deliver essential nutrients and signals throughout their long processes (Sharma et al., 2024). This ultimately leads to neuronal loss and brain atrophy, the shrinkage of brain tissue, observed in AD patients (Rivera et al., 2023).

AD manifests in two primary forms: early-onset familial AD and late-onset sporadic AD. Early-onset familial AD, constituting approximately 1% of cases, exhibits a compelling genetic link. Deterministic mutations in specific genes dramatically elevate the risk of developing the disease. APP, located on chromosome 21, harbors mutations that lead to increased production of Aβ peptides and therefore amyloid plaques (Wiseman et al., 2018). Presenilin 1 (PSEN1) and presenilin 2 (PSEN2), residing on chromosomes 14 and 1 respectively, encode crucial components of the gamma-secretase complex, an enzyme essential for APP processing and Aβ generation. Mutations in these genes disrupt gamma-secretase function, further augmenting Aβ production (De Strooper et al., 2012). In stark contrast, late-onset sporadic AD, encompassing around 99% of cases, presents with a more intricate genetic architecture. While genetics undoubtedly plays a role, it involves a confluence of multiple genes with subtler effects. Apolipoprotein E (APOE) stands out as the most prominent genetic risk factor for late-onset AD. Existing in three main allelic variants (ε2, ε3, and ε4), APOE ε4 is the strongest genetic susceptibility factor. Inheriting one copy of APOE ε4 increases an individual’s risk, whereas having two copies significantly elevates it. However, APOE ε4 does not guarantee the development of AD, highlighting the complex interplay between various genetic and environmental modulators of disease susceptibility (De Strooper et al., 2012). Recent advancements in genome-wide association studies have unveiled additional susceptibility loci, pointing towards a polygenic model for late-onset AD. These findings suggest that a combination of genetic variants, each with a modest effect size, collectively influences disease risk. Further exploration of these loci and their functional implications holds promise for uncovering novel therapeutic targets (Manna et al., 2023).

**Figure 1:**
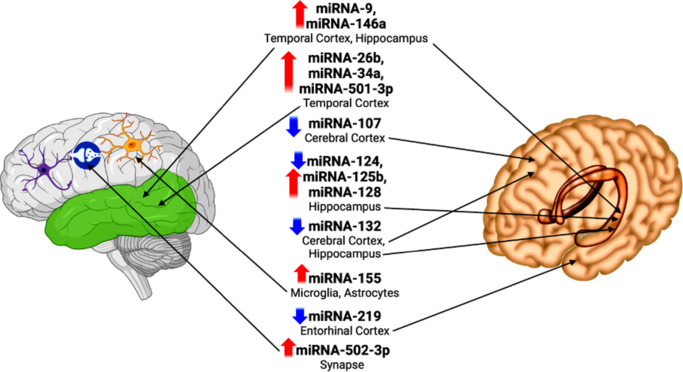
Potential brain-specific microRNAs (miRs) and their location within the brain regions. These miRs are dysregulated in Alzheimer’s disease (AD), some are upregulated and some of them are downregulated in AD relative to the control brain. Blue downward arrow (↓) indicates that the miRNA is downregulated in AD compared to healthy controls. Red upward arrow (↑) indicates that the miRNA is upregulated in AD relative to controls. Astrocytes are star-shaped glial cells depicted in orange on the left hemisphere and support neuronal function and modulate inflammation. Cerebral cortex is the outermost layer of the brain shown in light gray on the left and involved in cognition, decision-making, and memory. Entorhinal cortex is a medial temporal lobe region involved in memory and navigation and associated with miR-219. Hippocampus is a brain region shown in brown on the sagittal (right) brain; crucial for learning and memory formation. Microglia are immune cells of the central nervous system, shown in purple, and key regulators of neuroinflammation. miRs are small non-coding RNA molecules that regulate gene expression post-transcriptionally. Each label (e.g., miR-124 and miR-132) denotes a specific microRNA. Synapse is the junction between two neurons where neurotransmission occurs; miR-502-3p is localized here. Temporal cortex is a brain region shown in green on the left, involved in auditory processing and memory, and affected early in AD. Created with BioRender.com.

The relentless accumulation of these abnormal Aβ peptides and tau protein aggregates disrupts the delicate neuronal milieu, triggering a cascade of detrimental cellular processes. Aβ plaques are believed to disrupt synaptic function, the cornerstone of neuronal communication, by impeding neurotransmitter release and receptor activity. Furthermore, Aβ plaques can trigger chronic neuroinflammation, a self-perpetuating cycle of immune cell activation and glial reactivity, that further damages neurons and impedes repair mechanisms (Karisetty et al., 2020). NFTs, conversely, arise from the abnormal hyperphosphorylation of tau protein, a crucial component of the microtubule network that maintains neuronal structure and facilitates axonal transport, the vital process by which neurons deliver essential nutrients and signals throughout their long processes. Hyperphosphorylated tau proteins lose their functional capacity and aggregate into tangles, disrupting axonal transport and ultimately leading to neuronal dysfunction and death. The cumulative effects of Aβ plaque-induced synaptic dysfunction, chronic neuroinflammation, and NFT-mediated disruption of axonal transport culminate in a devastating cascade of cellular demise (Rivera et al., 2023). Mitochondrial dysfunction, a hallmark of AD, further compromises neuronal energy production and exacerbates cellular stress (Bose et al., 2024). This leads to the progressive loss of synapses, the dismantling of neuronal networks, and ultimately neuronal death (Dalal et al., 2024). This neurodegeneration is most prominent in brain regions critical for memory, thinking, and other cognitive functions, including the hippocampus and the entorhinal cortex (Sharma et al., 2024; **Figure 1**). As neurons succumb and brain tissue shrinks, brain atrophy occurs (Sharma et al., 2021).

## Search Strategy

A comprehensive literature search was conducted using the PubMed and Google Scholar databases via their respective institutional access platforms. The search was performed between June 2024 and March 2025. The following keywords were used in various combinations: “Alzheimer’s disease,” “microRNA,” “brain-specific microRNAs,” “miR-501-3p,” “miR-502-3p,” “neurodegeneration,” “synaptic dysfunction,” and “biomarkers.” The search included peer-reviewed primary research articles, review papers, and preclinical studies that examined the relationship between brain-specific miRNAs and AD pathophysiology, diagnostic potential, or therapeutic implications. No language restrictions were applied. Articles were included if they directly addressed the dysregulation or mechanistic role of brain-specific miRNAs in AD. Studies not related to AD or not specifically focusing on the miRNAs of interest were excluded.

## MicroRNAs

Micro ribonucleic acid, known as microRNA or miR, is a class of small, non-coding RNAs that play a critical role in regulating gene expression in various biological processes, including development, differentiation, and cellular homeostasis. These molecules, typically ranging from 19 to 25 nucleotides in length, function primarily as post-transcriptional regulators (Devara et al., 2023). Initially transcribed in the nucleus by RNA polymerase II as long primary miR transcripts, they undergo a series of enzymatic modifications by Drosha and Dicer, culminating in the formation of the mature, single-stranded miR (Bhaskaran and Mohan, 2014). Unlike protein-coding genes, miRs lack an open reading frame and do not translate into functional proteins. However, their impact on gene expression is substantial, achieved through two main mechanisms: translational repression and messenger RNA (mRNA) degradation (Prel et al., 2021). In the process of translational repression, mature miRs bind to the 3’ untranslated region (3’UTR) of target mRNAs. This interaction typically involves complementary base pairing between the miR seed sequence (nucleotides 2–8) and the target mRNA (Oliveto et al., 2017). The binding of the miR-mRNA complex recruits various protein factors, including the Argonaute protein family, which inhibits the translation of the mRNA into a functional protein (Valinezhad et al., 2014). In some instances, miR-mediated repression can lead to mRNA deadenylation and subsequent degradation, further silencing gene expression (Djuranovic et al., 2012). The expression and activity of miRs themselves are subjected to regulation by various factors, including histone modification and deoxyribonucleic acid (DNA) methylation (Malumbres, 2013). The involvement of miRs in various physiological processes underscores their potential role in the development and progression of numerous diseases, including neurodegenerative disorders such as AD (Li et al., 2023).

## Brain-Specific MicroRNAs

**Figure 2:**
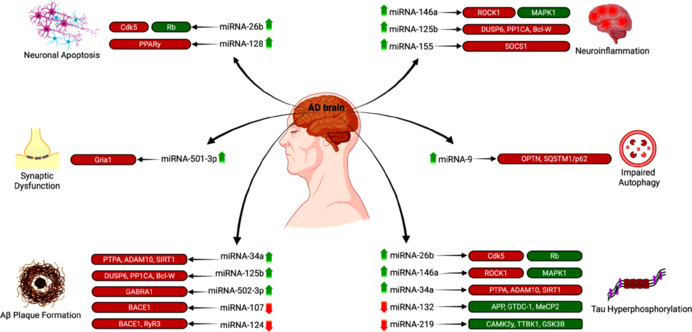
Brain-specific miRNAs and their impact on target gene(s). A green upward arrow (↑) next to a miRNA indicates that the miRNA is upregulated in AD brain tissue relative to control. A red downward arrow (↓) next to a miRNA indicates that the miRNA is downregulated in AD brain tissue compared to healthy controls. An arrow from a miRNA to a target gene in a green box indicates that the miRNA enhances or upregulates the expression of that gene. An arrow from a miRNA to a target gene in a red box indicates that the miRNA suppresses or downregulates the expression of that gene. Deregulation of these miRNAs alters their target genes, eventually affecting several cellular and biological processes in the brain and initiating AD progression. Aβ plaque formation refers to the accumulation of Aβ peptides forming amyloid plaques, a core feature of AD pathology. AD brain represents a patient with AD with the 6 downstream effects of dysregulated miRNA related to the condition. Gene targets represent specific mRNA/protein targets regulated by each miRNA (e.g., GSK3β, PPARγ, BACE1). Impaired Autophagy represents downstream effect resulting from dysregulation of autophagy-related genes such as *SQSTM1*/*p62* and *OPTN*. miRNAs (e.g., miRNA-9 and miRNA-132) are small non-coding RNAs that regulate gene expression post-transcriptionally. Neuronal Apoptosis refers to programmed cell death of neurons due to disrupted gene regulation and toxic accumulations. Neuroinflammation represents inflammation within the central nervous system, often driven by overactive immune responses (e.g., SOCS1 and PPARγ inhibition). Synaptic Dysfunction indicates disrupted neurotransmission caused by changes in genes such as *Gria1* and *GABRA1*. Tau hyperphosphorylation refers to abnormal phosphorylation of tau protein, contributing to neurofibrillary tangle formation in AD. Created with BioRender.com. AD: Alzheimer’s disease; Aβ: amyloid-beta; BACE1: β-site amyloid precursor protein cleaving enzyme 1; GSK3β: glycogen synthase kinase 3 beta; MiRNA: microRNAs; PPARγ: peroxisome proliferator-activated receptor gamma; SOCS1: suppressor of cytokine signaling 1; SQSTM1: sequestosome-1.

Within miR, a specific subset exhibits the unique characteristic of brain enrichment. These brain-specific miRs are predominantly expressed within the central nervous system (CNS), highlighting their potential significance in neuronal development, function, and potentially, disease (Reddy et al., 2017). The human brain harbors an array of neuronal cell types with distinct functions. This intricate organization necessitates a tightly regulated gene expression program to ensure proper development, differentiation, and maintenance of neuronal circuits. Brain-specific miRs contribute to this precise control by targeting a wide range of genes involved in numerous neurobiological processes (Zolboot et al., 2021). miRs are crucial for regulating the proliferation, migration, and differentiation of neural stem cells into mature neurons and glia. Specific brain-enriched miRs, such as miR-124, have been shown to play essential roles in this process of neural development (Zhang et al., 2023). The formation and refinement of synapses, the communication junctions between neurons, is a dynamic process known as synaptogenesis, which is critical for learning and memory (Dalal et al., 2024). Brain-specific miRs such as miR-132 and miR-128 are known to regulate genes involved in synaptic structure and plasticity (Hu and Li, 2017; **Figure 2**). The proper balance of neurotransmitters, chemical messengers between neurons, is essential for healthy brain function (Teleanu et al., 2022). Certain brain-enriched miRs, such as miR-25 and miR-185, target genes involved in neurotransmitter synthesis, release, and reuptake, such as SarcoEndoplasmic Reticulum ATPase (SERCA2), thus influencing neuronal communication and signaling (Thomas et al., 201). Myelination, the insulation of neuronal axons by myelin sheaths formed by glial cells, is crucial for efficient signal transmission (de Faria et al., 2019). Brain-specific miRs such as miR-214 and miR-9 regulate genes involved in this process by blocking the expression of myelin components including Myelin Associated Oligodendrocyte Basic Protein (Mobp) (Barca-Mayo and Lu, 2013). The mechanisms by which brain-specific miRs exert their regulatory influence are similar to those of other miRs. These miRs typically bind to the 3’UTR of target mRNAs, leading to either translational repression or mRNA degradation (Adlakha et al., 2014; **Table 1**). However, the specific repertoire of brain-enriched miRs and their target genes contribute to the unique regulatory landscape within the CNS (**Figure 1**). Understanding the expression patterns and functional roles of brain-specific miRs holds immense potential for understanding the complexities of nervous system function and dysfunction (Zolboot et al., 2021).

**Table 1:** Brain-specific microRNAs (miRs), their location, and relevance in human diseases

miRs	Brain-specific locations	Organisms	Chromosomal locations	Other diseases/disorders	References
miR-9	Putamen (basal ganglia), cerebellar hemisphere	Human	1q22, 5q14.3, 15q26.1	Cervical cancer, squamous cell carcinoma	Liu et al., 2020; GTEx Consortium, 2025
miR-26b	Cerebellar hemisphere, spinal cord (cervical c-1)	Human	2q35	Breast cancer, glioma	Huret et al., 2013; GTEx Consortium, 2025
miR-34a	Hippocampal neurons	Mouse	1p36	Atherosclerosis, ischemic stroke	Wei et al., 2019; Hu et al., 2020
miR-107	Tibial nerve, cerebellar hemisphere, basal ganglia	Human	10q23.31	Pancreatic cancer	Lee et al., 2009; GTEx Consortium, 2025
miR-124	Neurons	Human	8p23.1	Parkinson' disease, Huntington's disease	Kozuka et al., 2019; Han et al., 2020
miR-125b	Olfactory bulb, thalamus, hypothalamus	Mouse	11q24, 21q21	Colon cancer, hematopoietic tumor	Banzhaf-Strathmann and Edbauer, 2014; Hou et al., 2023
miR-128	Cerebellar hemisphere, caudate (basal ganglia)	Human	3p22	Non-small cell lung cancer	Cai et al., 2017; GTEx Consortium, 2025
miR-132	Cerebellar hemisphere, hippocampus, cerebellum	Human	17p13.3	Bipolar disorders, schizophrenia	Wanet et al., 2012; GTEx Consortium, 2025
miR-146a	Temporal lobe neocortex, hippocampus	Mouse	5q33.3	Coronary artery disease, kidney fibrosis	Lukiw et al., 2008; Paterson and Kriegel, 2017
miR-155	Microglia, astrocytes, neuronal nuclei	Mouse	21q21.3	Breast cancer, lung cancer	Zingale et al., 2021; Landeros et al., 2022
miR-219	Hypothalamus, anterior cingulate cortex (BA24)	Human	9q131,154,291-131,155,789	Multiple sclerosis, leukodystrophies	Wang et al., 2017; GTEx Consortium, 2025; No author listed, 2025
miR-501-3p	Frontal cortex (BA9), cerebellum, cortex	Human	Xp11	Schizophrenia, dementia	He et al., 2021; Devara et al., 2023; GTEx Consortium, 2025
mir-502-3p	Cerebellar hemisphere, cervical c-1 spinal cord	Human	Xp50,014,598-50,014,683	Diabetes type 2, osteoporosis	Devara et al., 2023; GTEx Consortium, 2025

## MicroRNAs and Alzheimer’s Disease

This regulatory process of miR by inhibiting their translation or promoting their degradation positions it as key players influencing a vast array of cellular processes relevant to AD. The involvement of miRs in AD unfolds through a captivating paradox. Certain miRs appear to act as detrimental protagonists, actively promoting neurodegeneration. These miRs achieve their effects by targeting genes critical for neuronal health and function. One such example involves genes implicated in synaptic function, the cornerstone of neuronal communication. By silencing these genes, these miRs disrupt the communication between neurons, a hallmark dysfunction observed in AD such as dysregulated synaptic excitability and synaptic ATP production (Kumar and Reddy, 2020). Furthermore, some miRs target genes responsible for regulating tau protein phosphorylation. In its healthy state, tau protein maintains the structural integrity of neuronal processes. These certain miRs can exacerbate this process by dysregulating tau phosphorylation by targeting kinases and phosphatases, further contributing to neurodegeneration (Praticò, 2020; **Figure 2**).

Furthermore, some miRs appear to inadvertently silence genes that normally protect neurons from damage. By silencing these neuroprotective genes, these miRs leave neurons vulnerable to the cellular stresses that contribute to AD. Conversely, certain miRs emerge as defenders against neurodegeneration. These miRs target genes that can promote Aβ plaque clearance and even reverse its neuronal damage. They can do this by targeting genes responsible for either Aβ production or its breakdown, potentially influencing the rate of plaque buildup in the brain (Rivera et al., 2023). Moreover, certain miRs act as shields, enhancing the resilience of neurons against various cellular stresses. By supporting neuronal defenses, these miRs promote neuronal survival and function. Finally, miRs can modulate the activity of microglia, the brain’s immune cells, influencing the inflammatory response in AD. Microglia play a crucial role in both clearing debris and inflammation. miRs can fine-tune microglial activity, promoting a less inflammatory response and creating a more neuroprotective environment in the brain (Pratico, 2020; **Table 2**).

**Table 2:** Details of brain-specific miRs in AD

miRs	Brain-specific locations	Status in AD	Models	Target gene(s)	Outcomes	Proposed benefits	Biomarker/therapeutic challenges	References
miR-9	Temporal cortex, hippocampus	Upregulated	Mouse	OPTN, SQSTM1/p62	Impaired autophagy, Aβ plaque, and NFT buildup	Biomarker, therapeutic	Lack of delivery precision, off-target effects, need for cell-specific targeting	Chen et al., 2021
miR-26b	Temporal cortex	Upregulated	Human postmortem brain	Rb, Cdk5	Postmitotic neuron cell cycle re-entry, Cdk5 mis localization	Therapeutic	Potential for cell cycle dysregulation, complex downstream effects	Absalon et al., 2013
miR-34a	Temporal cortex	Upregulated	Mouse	PTPA, ADA10, SIRT1	Tau hyperphosphorylation, Aβ accumulation, NR2A loss	Therapeutic	Challenge in modulating multiple pathways simultaneously	Sarkar et al., 2016; Sarkar et al., 2019; Wei et al., 2019
miR-107	Cerebral cortical laminas	Downregulated	Human postmortem brain	BACE1	Higher BACE1 expression, Aβ plaque formation	Therapeutic	Difficulty in restoring expression without affecting other tissues	Zhu et al., 2004; Wang et al., 2008; Sun et al., 2020
miR-124	Neurons	Downregulated	Human postmortem brain, mouse	RyR3, BACE1	Calcium signaling dysregulation, increase Aβ plaque	Therapeutic	Stability and targeted delivery limitations	Fang et al., 2012; Liu et al., 2014; Han et al., 2020; Vaz et al., 2021
miR-125b	Primary hippocampal neurons	Upregulated	Mouse	DUSP6, PP1CA, Bcl-W	Promote APP, Aβ peptide, suppress SphK1	Biomarker, therapeutic	Potential toxicity, pro-inflammatory outcomes	Banzhaf-Strathmann et al., 2014; Hong et al., 2017; Jin et al., 2018
miR-128	Hippocampus, cortex	Upregulated	Human postmortem brain	PPARγ	Increase pro-inflammatory mediators, neuroinflammation	Therapeutic	Anti-inflammatory targeting complexity	Li et al., 2023
miR-132	Cerebral cortex, hippocampus	Downregulated	Mouse, rat	APP, GTDC-1, MeCP2	Produce Aβ, GTDC1/CDK5 signaling, tau phosphorylation	Biomarker, therapeutic	Challenge in restoring physiological levels and synaptic function	Wanet et al., 2012; Zhang and Bian, 2021;
miR-146a	Temporal lobe, hippocampus	Upregulated	Mouse	ROCK1, MAPK	Reduce p-PTEN levels, activate p-p38 MAPK signaling	Therapeutic	MAPK pathway involvement complicates specificity	Wang et al., 2016; Zhan-Kiang et al., 2023
miR-155	Microglia	Upregulated	Mouse	SOCS1	Activates Th1 and Th17 cells, neuroinflammation	Therapeutic	Risk of excessive immune modulation	Song and Li, 2015; Landeros et al., 2022
miR-219	Entorhinal cortex	Downregulated	Human postmortem brain	CAMK2γ, TTBK1, GSK3β	Tau hyperphosphorylation, myelin degeneration	Therapeutic	Therapy needs to avoid demyelination and preserve signaling	Santa-Maria et al., 2015; Arnes et al., 2019; Li et al., 2019
miR-501-3p	Temporal cortex	Upregulated	Human postmortem brain, mouse	Gria1	Decrease GluA1 expression, synaptic plasticity deficits	Biomarker, therapeutic	Synaptic targeting complexity, off-target neuronal effects	He et al., 2021; Devara et al., 2023;
miR-502-3p	Synapses	Upregulated	Human postmortem brain, mouse	GABRA1	Reduce GABRα1 protein/GABAergic synaptic signaling	Biomarker, therapeutic	Interference with GABAergic tone could worsen cognition	Devara et al., 2023

AD: Alzheimer’s disease; APP: amyloid precursor protein; Aβ: amyloid-beta; BACE1: β-site amyloid precursor protein cleaving enzyme 1; Bcl-W: Bcl‐2‐like protein 2; CAMK2γ: calcium/calmodulin-dependent protein kinase 2 gamma subunit; DUSP6: dual‐specific phosphatase 6; GABRα1: gamma-aminobutyric acid type A receptor subunit alpha1; GluA1: α-amino-3-hydroxy-5-methyl-4-isoxazolepropionic acid (AMPA) receptor subunit glutamate receptor 1; GSK3β: glycogen synthase kinase 3 beta; GTDC-1: glycosyltransferase like domain containing 1; MAPK: mitogen-activated protein kinase; miRs: microRNAs; PP1CA: protein phosphatase 1 catalytic subunit alpha isoform; PPARγ: peroxisome proliferator-activated receptor gamma; RyR3: ryanodine receptor 3; SIRT1: sirtuin 1; SOCS1: suppressor of cytokine signaling 1; SQSTM1: sequestosome-1; TTBK1: Tau tubulin kinase 1.

This intricate dynamic between various miRs underscores the multifaceted role they play in AD. Understanding the specific functions of these various miRs is paramount to gaining a deeper understanding of the disease process and its potential therapeutic manipulation. The promising potential of miRs as therapeutic targets in AD comes from several compelling advantages. Their remarkable specificity, targeting specific mRNAs with precision, offers the potential to minimize unwanted side effects associated with conventional therapies. Additionally, the accessibility of miRs in bodily fluids, including blood and cerebrospinal fluid (CSF), makes them potentially useful as biomarkers. By analyzing miR profiles in these fluids, researchers might be able to diagnose AD earlier or even monitor disease progression (Kumar and Reddy, 2021). The future of miR research in AD is full of promise. Scientists are actively engaged in identifying disease-associated miRs and studying their precise functions. This ever-expanding knowledge base paves the way for the development of miR-based therapies for AD. The ability to manipulate miR expression has the potential to revolutionize AD treatment in various ways. One way is by possibly slowing or preventing neurodegeneration. By promoting neuronal health and function through targeted miR modulation, researchers hope to slow or even prevent the relentless neurodegeneration that characterizes AD. Aβ plaque burden can also be reduced by enhancing Aβ clearance or inhibiting its production through strategic miR manipulation (Li et al., 2023). Furthermore, by fine-tuning the inflammatory response in the brain through miR manipulation, researchers hope to create a more neuroprotective environment, fostering neuronal survival and function (Ma et al., 2019). While challenges remain, such as ensuring the safe and effective delivery of miR-based therapies to the brain, the potential of miRs in AD is undeniable. By unraveling the complex web of miR regulation, researchers are poised to translate this exciting area of research into novel therapeutic strategies for combating this devastating neurodegenerative disease.

### MicroRNA-9 and Alzheimer’s disease

Encoded by three genes located on Chromosome 1q22, 5q14.3, and 15q26.1, miR-9 has been seen to play a versatile role in cancers such as cervical cancer and squamous cell carcinoma (Liu et al., 2020; **Table 1**). miR-9 also exhibits a dynamic and potentially stage-dependent role in AD pathogenesis, transitioning from a neuroprotective factor in the early stages to a disease-promoting element in later stages (Miya Shaik et al., 2018). A previous study employing Aβ plaque-burdened mouse models of AD reveal a significant downregulation of miR-9 in primary hippocampal neurons during early disease progression (Schonrock et al., 2010; **Figure 1**). This downregulation is accompanied by impaired autophagy, a cellular waste disposal mechanism critical for maintaining neuronal health (Liénard et al., 2024). Mechanistically, miR-9 directly targets optineurin, an autophagy receptor protein, for degradation (**Figure 2**). By inhibiting miR-9 with antagomirs, researchers were able to restore optineurin levels and enhance autophagic activity in these late-stage models. This functional restoration translated into a robust behavioral improvement, with mice exhibiting enhanced memory, improved motor function, and a reduction in brain Aβ plaque burden. These findings suggest that miR-9 inhibition may serve as a potential therapeutic strategy to promote autophagy and mitigate AD pathology (Chen et al., 2021). Further supporting the link between miR-9 and autophagy, the study demonstrated a concomitant upregulation of sequestosome-1 (SQSTM1) and p62, another autophagy adaptor protein, upon miR-9 inhibition. SQSTM1 accumulation is a hallmark of impaired autophagy, and its elevation alongside restored optineurin levels strengthens the argument for the role of miR-9 in regulating the autophagic machinery during AD progression (Subramanian et al., 2021). Since autophagy is the process in which damaged organelles and protein aggregates are broken down, impaired autophagy caused by miR-9 causes the buildup of Aβ plaque and NFTs.

Adding to the preclinical findings, recent human cohort studies have provided compelling evidence that miR-9 levels are significantly altered in late-onset AD. A study involving whole blood samples from 74 older women (> 55 years), all APOE carriers, reported a median 3-fold decrease in miR-9 levels in patients with probable AD compared to cognitively intact controls (*P* = 0.001). This association remained significant even after adjusting for age and APOE genotype (Souza et al., 2020). The use of real-time PCR to quantify miR-9 in whole blood strengthens the case for its utility as a non-invasive, blood-based biomarker. The same study confirmed that reduced miR-9 levels in peripheral blood are consistent with previous findings of decreased miR-9 in brain, CSF, plasma, and serum samples of AD patients. Importantly, miR-9 has been shown to regulate key genes involved in AD pathogenesis, including BACE1, which drives Aβ production, and sirtuin 1 (SIRT1), a neuroprotective factor that prevents tau hyperphosphorylation (Souza et al., 2020). The convergence of these findings across multiple human biofluids highlights miR-9’s potential role not just in diagnosis but also in the pathophysiological mechanisms of AD. Moreover, the study suggests that miR-9 can traverse the blood–brain barrier (BBB) via exosomes or other transport vesicles, further supporting the idea that peripheral blood levels could reflect central nervous system activity. This transportability adds clinical value to miR-9 as a biomarker, especially when compared to current CSF-based biomarkers that require invasive lumbar puncture. The observed downregulation in the early stages stands in stark contrast to the upregulation in the temporal cortex and hippocampus observed later, highlighting a potential shift in miR-9’s function as the disease progresses and suggesting its expression might serve as a biomarker to differentiate between early and late-stage AD (Chen et al., 2021; **Table 2**).

### MicroRNA-26b and Alzheimer’s disease

MiR-26b is encoded by the genes found on Chromosome 2q35 and has been established to play a role in cancers such as breast cancer and glioma development (Huret et al., 2013; **Table 1**). Studies have demonstrated a significant upregulation of miR-26b in the temporal cortex, a brain region particularly vulnerable to AD pathology, when comparing human subjects diagnosed with AD or mild cognitive impairment (MCI) to healthy controls (**Figure 1**). This regional specificity suggests a targeted effect of miR-26b on vulnerable neuronal populations within the temporal lobe, potentially contributing to the characteristic memory decline observed in AD patients (Liu et al., 2016). Recent research has shed light on its potential role in disrupting the delicate homeostasis and tightly regulated cell cycle within postmitotic neurons, a population of terminally differentiated neurons that cease cell division in the mature brain and are critically affected in AD (Absalon et al., 2013). The retinoblastoma protein (Rb) acts as a crucial cell cycle inhibitor, ensuring the growth arrest of postmitotic neurons (Omais et al., 2022). Research suggests that miR-26b directly targets Rb, leading to its downregulation. This reduction in Rb levels disrupts the normal cell cycle arrest, triggering an abnormal re-entry into the cell cycle in these postmitotic neurons (Zhang et al., 2020). This unscheduled proliferation is further substantiated by the observed increase in expression of cell cycle markers such as antigen Kiel 67, proliferating cell nuclear antigen, and cyclin E1, hallmarks of active cell division. The abnormal cell cycle re-entry triggered by miR-26b appears to culminate in increased neuronal apoptosis, further exacerbating the neurodegeneration observed in AD (Absalon et al., 2013). Furthermore, cyclin-dependent kinase 5 (Cdk5), a kinase implicated in tau phosphorylation, exhibits altered cellular localization upon miR-26b upregulation (Zhang et al., 2021). This mislocalization of Cdk5 is hypothesized to contribute to the hyperphosphorylation of tau protein, a crucial step in the formation of NFTs (Zheng et al., 2005; **Figure 2**). These findings unveil a novel mechanism by which miR-26b might contribute to AD pathogenesis. By targeting Rb and disrupting the cell cycle machinery within postmitotic neurons, miR-26b appears to trigger a cascade of detrimental events, including aberrant proliferation, Cdk5 mislocalization, tau hyperphosphorylation, and ultimately neuronal death. These findings propose a novel mechanism by which miR-26b might contribute to AD pathogenesis. Inhibiting miR-26b could potentially prevent the deleterious effects observed in these studies (**Table 2**). Specifically, by blocking the ability of miR-26b to induce cell cycle re-entry and disrupt Cdk5 localization, such an approach might offer a neuroprotective strategy for AD (Absalon et al., 2013).

### MicroRNA-34a and Alzheimer’s disease

MiR-34a contributes to the susceptibility of various diseases and disorders including atherosclerosis and ischemic stroke as it encoded by the genes found in the Chromosome 1p36 (Wei et al., 2019; **Table 1**). Researchers investigated the role of miR-34a, which is found to be upregulated in the temporal cortex, in AD using a mouse model engineered to overexpress miR-34a (Sarkar et al., 2016; **Figure 1**). Using miR-34a Target Scan analysis, researchers identified protein phosphatase 2A activator (PTPA), which helps regulate tau phosphorylation, as a potential target of miR-34a. Western blot analysis confirmed that PTPA protein levels were significantly reduced in brain regions of mice overexpressing miR-34a (Sarkar et al., 2019). This suggests that miR-34a might promote tau hyperphosphorylation by reducing PTPA levels by impairing the activation of PP2A, a critical phosphatase involved in dephosphorylating tau. PTPA activates PP2A by reducing the inhibitory phosphorylation at tyrosine-307 (P-PP2AC) of the PP2A catalytic subunit (PP2AC) and increasing its methylation at leucine-309, promoting its active form. This activation of PP2A leads to dephosphorylation of tau at multiple sites, including Ser262, Ser396, Ser404, Ser422, and Ser198/199/202. When PTPA levels are reduced, as seen in AD brains and transgenic mouse models, PP2A becomes less activated. This is due to the increased inhibitory phosphorylation of PP2AC at tyrosine-307 and a decrease in PP2AC methylation. As a result, the ability of PP2A to dephosphorylate tau is diminished, leading to enhanced tau phosphorylation. Thus, the reduction of PTPA disrupts PP2A activation, which in turn contributes to tau hyperphosphorylation, a key feature in AD pathology (Luo et al., 2013). Another line of investigation focused on Aβ. Sirtuin 1 (SIRT1) and disintegrin and metalloproteinase 10 (ADAM10) are proteins involved in the non-amyloidogenic processing of APP, which helps prevent Aβ formation. Target Scan analysis again predicted that miR-34a might target both SIRT1 and ADAM10. Western blot analysis confirmed a significant reduction of ADAM10 protein levels, and therefore accumulation of intraneuronal Aβ, in various brain regions of mice overexpressing miR-34a (Sarkar et al., 2016). There was also a decrease in NR2A-containing N-methyl-D-aspartate receptor, due to a decline in SIRT1 targeted by miR-34a, contributing to cognitive decline and memory impairment (McQuail et al., 2016). Furthermore, immunohistochemistry experiments revealed a substantial increase in Aβ deposits within the neurons of these mice. Interestingly, the Aβ staining appeared primarily intracellular, suggesting an early stage in Aβ accumulation (**Figure 2**). The study also observed a remarkably rapid decline in cognitive function within 1–2 months of miR-34a overexpression, significantly faster than the onset observed in typical AD mouse models (Sarkar et al., 2019). This rapid development of AD-like features highlights the potential of this model for investigating the very early stages of AD pathology.

### MicroRNA-107 and Alzheimer’s disease

MiR-107 is encoded by the genes found on Chromosome 10q23.31 and has emerged as a factor in various diseases and cancers, namely pancreatic cancer (Zhou et al., 2007; **Table 1**). miR-107 has also emerged as a potential regulator of β-site amyloid precursor protein-cleaving enzyme 1 (BACE1), a key enzyme involved in the production of beta-amyloid plaques, a hallmark pathology of AD (Zhao et al., 2007). This connection has been investigated by analyzing mRNA levels of BACE1 and miR-107 in the temporal cortex of individuals with varying degrees of AD pathology, including non-demented, MCI, and AD (**Figure 1**). The experiment observed a statistically significant inverse correlation of cases with lower miR-107 levels in the cerebral cortical laminas, typically those with more severe AD pathology, exhibited higher levels of BACE1 mRNA (Wang et al., 2008). To understand which miRs are found during brain states, researchers employed cross-correlation analysis using microarrays (Babak et al., 2004). Additionally, bioinformatics analysis predicted that miR-107 might target up to five specific sites on the 3’UTR of BACE1 mRNA, potentially regulating its translation into protein. Both the microarrays and bioinformatics analysis directly demonstrated that miR-107 regulates BACE1 expression by targeting at least one specific region within its 3’UTR. These findings collectively suggest that a decrease in neuronal miR-107 levels contributes to AD development. Lower miR-107 allows for increased BACE1 production, which in turn leads to the formation of more Aβ plaques (Wang et al., 2008; **Figure 2**). *In situ* hybridization experiments provided further support by confirming that miR-107 is primarily expressed in neurons within the cerebral cortex, positioning it to directly regulate BACE1 in these critical cells (Wang et al., 2023). Interestingly, miR-107 expression appeared to be reduced early in the pathological progression of AD, suggesting its potential as a target for therapeutic intervention strategies aimed at preventing or slowing disease progression (Nelson and Wang, 2010).

Clinical studies further support the biomarker potential of miR-107. In a human cohort study involving peripheral blood profiling, miR-107 was significantly downregulated in both AD and MCI patients compared to cognitively normal controls across different ethnic populations, including Chinese and Caucasian cohorts (Wu et al., 2016). This downregulation was consistently observed in two independent studies and was associated with high diagnostic performance. In the study, miR-107 demonstrated a sensitivity of 90% and specificity of 78% for detecting AD from plasma samples, indicating strong discriminatory power. The same study also showed that miR-107 was downregulated in MCI, with an even higher sensitivity of 98% and specificity of 83%, suggesting its potential as an early-stage biomarker before full-blown AD manifests. These results position miR-107 as not only a mechanistic regulator of Aβ pathology through BACE1 but also a promising peripheral diagnostic tool, particularly due to its accessibility via non-invasive blood-based assays (**Table 2**). Compared to conventional AD diagnostics like CSF Aβ/tau measurements or PET imaging, which are either invasive or expensive, miR-107 offers a cost-effective and scalable alternative with clinically meaningful sensitivity and specificity.

### MicroRNA-124 and Alzheimer’s disease

Encoded by genes found on the Chromosome 8p23.1 region, miR-124 has been associated with impaired prepulse inhibition, methamphetamine-induced hyperactivity, and social deficits in mice (Kozuka et al., 2019; **Table 1**). It is particularly abundant in neurons, highlighting its crucial role in neuronal function (Vaz et al., 2021). Studies have shown that miR-124 expression levels decrease in the hippocampus of AD patients compared to healthy individuals (**Figure 1**). This downregulation of miR-124 has been observed in both postmortem human brain tissue and animal models of AD, such as mice (Han et al., 2020). The decrease in miR-124 function is believed to contribute to the pathological processes underlying AD through its interaction with specific genes. miR-124 has been shown to target the ryanodine receptor 3 (*RyR3*) gene because when miR-124 levels are low, RyR3 expression increases (Liu et al., 2022). RyR3 gene plays a role in calcium signaling pathways within neurons and regulates the function of synapse plasticity, neuronal viability, and cognitive functions (Ali et al., 2023). The dysregulation of calcium signaling due to elevated RyR3 is thought to contribute to increased amyloid plaque formation and loss of hippocampal synaptic markers and neuronal deterioration, hallmark features of AD (Liu et al., 2014). A study utilizing pheochromocytoma (PC12) cells and primary cultured hippocampal neurons showed an inverse relationship between miR-124 and BACE1, with reduced miR-124 resulting in upregulated BACE1. Since BACE1 is the rate-limiting enzyme in Aβ generation, upregulation of BACE1 by miR-124 could potentially increase Aβ levels. Furthermore, the study demonstrated that Aβ-induced neuronal toxicity was mitigated by miR-124 overexpression, suggesting a neuroprotective function of miR-124 (Fang et al., 2012; **Table 2**). It may be able to protect neurons by targeting proteins like protein tyrosine phosphatase non-receptor type 1 (PTPN1), which when downregulated, contributes to synaptic abnormalities and memory decline by impairing the glutamate receptor 2 membrane insertion (Kumar and Reddy, 2020; **Figure 2**). Researchers are actively exploring methods to increase miR-124 levels in the brain, such as utilizing adeno-associated viruses (AAV) for targeted delivery of miR-124 (Zhou et al., 2019).

### MicroRNA-125b and Alzheimer’s disease

MiR-125b, with miR-125b-1 encoded by genes on Chromosome 11q24 and miR-125b-2 encoded by genes on Chromosome 21q21, is a miR positively correlated with various tumors such as colon cancer and hematopoietic tumors (Banzhaf-Strathmann and Edbauer, 2014; **Table 1**). To explore the significantly higher levels of miR-125b in primary hippocampal neurons in AD patients, scientists used an in vitro model employing mouse neuroblastoma cells engineered to mimic aspects of AD (**Figure 1**). They manipulated these cells to overexpress miR-125b and observed several concerning effects such as reduced cell proliferation and increased apoptosis due to its targeting of Bcl‐2‐like protein 2 (Bcl-W), dual‐specific phosphatase 6 (DUSP6), and protein phosphatase 1 catalytic subunit alpha isoform (PPP1CA) and therefore the downregulation of those genes (Banzhaf-Strathmann et al., 2014). Enhanced inflammation was also observed through the enhanced expression of inflammatory factors such as tumor necrosis factor-alpha (TNF-α), interleukin-1 beta (IL-1β), and interleukin 6 (IL-6). Oxidative stress was also increased with the overexpression of miR-125b which was associated with inhibited superoxide dismutase (SOD) levels and increased malondialdehyde (MDA) levels (Jin et al., 2018). These observations suggest that miR-125b might contribute to neuronal death, a hallmark of AD, and worsen inflammatory and oxidative stress processes that are known to promote AD progression (Tönnies and Trushina, 2017). Moreover, western blotting revealed that miR-125b overexpression increased the production of APP, BACE1, phosphorylated extracellular signal-related kinases (p-ERK) protein expression, and Tau1 protein levels (Jin et al., 2018). These are key pathological features of AD because abnormal processing of APP by BACE1 leads to plaque buildup, while Tau protein hyperphosphorylation forms tangles (Wen et al., 2022). Enzyme-linked immunosorbent assay (ELISA) analysis also detected promoted Aβ peptide production with increased miR-125b levels (Jin et al., 2018). This is significant because Aβ peptide production is a major factor in the cerebrovascular lesions and neurotoxicity observed in AD (Askarova et al., 2013). Interestingly, the study also found that miR-125b overexpression suppressed a protein called Sphingosine kinase 1 (SphK1) (Jin et al., 2018; **Table 2**). Increase in SphK1 has been seen to improve learning and memory while also reducing the deposition of amyloid proteins in APP/presenilin 1 (PS1) transgenic mice with AD (Li et al., 2017; **Figure 2**). These findings support the theory that miR-125b contributes to the development of AD and that its targeting can be a potential biomarker and therapeutic approach.

### MicroRNA-128 and Alzheimer’s disease

MiR-128, a brain-enriched microRNA encoded by the *miR-128-1* and *miR-128-2* loci, has gained considerable attention as a dynamic and multifaceted regulator in the pathophysiology of AD (**Table 1**). Unlike many microRNAs that exhibit relatively linear and consistent functional profiles, miR-128 is characterized by a paradoxical role, displaying both neuroprotective and potentially neurotoxic effects depending on the cellular context, disease stage, and expression threshold. Its strategic enrichment in critical brain regions such as the hippocampus and cerebral cortex—areas intimately involved in memory, learning, and executive function—positions miR-128 to play a pivotal role in modulating neuronal health and disease progression (**Figure 1**). The existing literature on miR-128 presents conflicting data regarding its expression patterns in AD, further underscoring its complex biological nature. Initial studies reported a downregulation of miR-128 in AD brains. This reduction was linked to deleterious outcomes, including increased glycogen synthase kinase 3 beta (GSK3β) activity, elevated tau phosphorylation, and enhanced production of Aβ peptides (Li et al., 2023). These findings suggested that diminished miR-128 levels compromise neuronal defenses and contribute to the early initiation of AD pathology. However, more recent studies utilizing postmortem samples of cortical and hippocampal tissues from AD patients have revealed an opposing trend—namely, an upregulation of miR-128 in comparison to age-matched cognitively normal individuals. This elevated expression has been interpreted by some researchers as a compensatory response by neurons or glial cells attempting to counteract ongoing neurodegenerative damage (Zhang et al., 2021; **Figure 2**). Nevertheless, such compensation may become maladaptive over time, leading to unintended consequences that could exacerbate disease progression.

These contradictory observations have given rise to two primary hypotheses. One posits that the initial downregulation of miR-128 weakens neuroprotective mechanisms, thereby facilitating the onset and early spread of AD pathology. The second hypothesis suggests that upregulation of miR-128 in later stages may reflect a homeostatic response that ultimately fails to restore cellular equilibrium, possibly due to dysregulated feedback loops or overactivation of certain signaling pathways. Importantly, these hypotheses are not mutually exclusive. Instead, they reflect the nuanced reality that miR-128 regulation is highly dependent on brain region, cellular origin (neuronal *versus* glial), and the temporal evolution of disease (Li et al., 2023). This dynamic expression pattern likely mirrors broader stress-response states within the brain as it attempts to adapt to the progressive burden of AD pathology. Functionally, miR-128 exerts regulatory control over multiple molecular cascades that are central to AD development and progression. One of the most well-characterized roles of miR-128 involves its inhibition of GSK3β, a serine/threonine kinase known to promote tau hyperphosphorylation at multiple pathological sites. miR-128 achieves this by directly binding to the 3′ untranslated region (3′ UTR) of *GSK3*β mRNA, thereby repressing its translation and reducing overall protein levels (Decressac et al., 2013). The resulting decrease in GSK3β activity leads to diminished tau phosphorylation and a subsequent reduction in neurofibrillary tangle formation, suggesting a strong neuroprotective mechanism by which miR-128 limits tau pathology (Li et al., 2023).

Beyond its influence on tau-related pathways, miR-128 also plays a critical role in modulating Aβ metabolism. It does so by targeting and suppressing the expression of APP-binding protein 2 (APPBP2) and the pro-inflammatory transcription factor nuclear factor kappa B (NF-κB). APPBP2 is known to stabilize APP at the neuronal cell membrane, thereby enhancing its cleavage into neurotoxic Aβ peptides. By inhibiting APPBP2 and NF-κB, miR-128 reduces APP stability and downregulates inflammatory signals that promote amyloidogenic processing. These effects culminate in decreased Aβ production and deposition, further supporting the potential of miR-128 as a neuroprotective agent in AD (Geng et al., 2018). In addition to suppressing Aβ production, miR-128 contributes to the clearance of toxic protein aggregates through its regulation of autophagy—a vital cellular process responsible for degrading misfolded proteins, damaged organelles, and pathological aggregates such as Aβ plaques. miR-128 enhances autophagic flux by inhibiting the mammalian target of rapamycin (mTOR), a central negative regulator of autophagy (Decressac et al., 2013). Inhibition of mTOR by miR-128 leads to increased activation of the autophagic pathway, thereby facilitating the removal of toxic cellular debris and alleviating proteostatic stress within neurons.

However, while these pathways highlight the beneficial aspects of miR-128, there is growing recognition that its overexpression may have deleterious consequences, particularly through the suppression of peroxisome proliferator-activated receptor gamma (PPARγ). PPARγ is a nuclear transcription factor known for its anti-inflammatory, antioxidative, and neuroprotective functions. It plays a critical role in modulating glial activation, cytokine production, and oxidative stress response (Geng et al., 2018). Elevated levels of miR-128 can inhibit PPARγ expression, leading to exacerbation of chronic neuroinflammation, reactive gliosis, and oxidative damage—hallmarks of neurodegenerative progression in AD (**Figure 2**). This dualistic behavior illustrates miR-128’s complex functionality, in which it may reduce amyloid and tau burden while simultaneously impairing immune homeostasis and exacerbating inflammatory damage (Villapol, 2018). Several limitations within the current research landscape impede the full translational potential of miR-128. A significant constraint is the frequent reliance on bulk tissue analysis in both human and animal studies. Such approaches obscure the underlying heterogeneity of cell types within the brain and fail to capture the distinct expression and function of miR-128 in neurons versus glial cells. Consequently, it remains unclear whether the observed changes in miR-128 expression in AD are primarily driven by neuronal adaptation, glial dysfunction, or an interplay between the two. Furthermore, most studies lack longitudinal data, making it difficult to determine how miR-128 expression evolves across different stages of AD or how it responds to therapeutic interventions.

Another critical unanswered question concerns the dose-dependent effects of miR-128. While low-to-moderate levels of expression may yield beneficial outcomes through the regulation of GSK3β and mTOR, excessive overexpression may disrupt cellular homeostasis by suppressing essential protective factors such as PPARγ or by producing unintended off-target effects. This threshold-dependent behavior has not been systematically studied and warrants further investigation using titration-based approaches in disease-relevant *in vivo* models. Additionally, the mechanisms underlying miR-128’s transport and release remain poorly characterized. It is currently unknown whether miR-128 is predominantly retained within cells or secreted via extracellular vesicles (EVs) such as exosomes. Determining whether neurons, astrocytes, or microglia are the principal sources of miR-128 expression and EV packaging will be critical for its development as a reliable and minimally invasive biomarker. If miR-128 is selectively enriched in EVs, its levels in cerebrospinal fluid or plasma-derived exosomes could serve as a sensitive indicator of disease stage or therapeutic response. Taken together, these findings establish miR-128 as a strategically vital yet mechanistically complex regulator of AD pathology. Its central involvement in tau phosphorylation, Aβ production, and clearance, autophagic activity, and neuroinflammation underscores its potential as both a therapeutic target and a diagnostic biomarker. The conflicting evidence regarding its precise role in AD should not be interpreted as contradictory, but rather as a reflection of the biological complexity inherent to miRNA-mediated regulation. Variations in disease stage, cellular context, expression thresholds, and methodological approaches all contribute to these divergent findings. Looking ahead, the most promising strategies for targeting miR-128 will likely involve precision modulation rather than global upregulation or inhibition. Therapeutic approaches could include the use of antisense oligonucleotides, CRISPR-based genome editing, or small molecules that specifically enhance beneficial interactions—such as the inhibition of GSK3β and mTOR—while mitigating harmful effects, such as PPARγ repression (**Table 2**). Concurrently, the development of miR-128 as a biomarker will require high-resolution, spatiotemporal datasets derived from single-cell RNA and miRNA sequencing, spatial transcriptomics, and longitudinal studies in both human patients and animal models.

In conclusion, miR-128 is not a simple binary regulator of AD pathology but rather a context-dependent, dual-role microRNA whose effects vary based on its spatial distribution, temporal expression, and dosage. Unlocking the full therapeutic and diagnostic potential of miR-128 will depend on resolving these outstanding questions—an effort that could ultimately pave the way for precision medicine approaches tailored to individual patients and specific stages of Alzheimer’s disease.

### MicroRNA-132 and Alzheimer’s disease

MiR-132 is known to regulate axon, dendritic, and spinal maturation in response to multiple signaling pathways. Recent research highlights its neuroprotective properties achieved through regulating a network of genes (El Fatimy et al., 2018). Unfortunately, miR-132 expression is significantly downregulated in the cerebral cortex and hippocampus of AD patients (Qian et al., 2017; **Figure 1**). Utilizing *in vivo* mouse and rat AD models as well as *in vitro* models, this deficiency has been found to disrupt its ability to safeguard neurons, contributing to the hallmarks of AD (Zhang and Bian, 2021). One key target of miR-132 is APP, which undergoes cleavage to generate Aβ peptides and therefore its accumulation in plaques. miR-132 directly binds to the APP mRNA, suppressing its translation and thereby reducing Aβ production. This means that the miR-132 deficiency observed in AD enhances Aβ production (Hernandez-Rapp et al., 2016). Additionally, miR-132 regulates genes involved in tau protein phosphorylation, another hallmark of AD. By targeting the glycosyltransferase like domain containing 1 (*GTDC-1*) gene and enzymes like GSK-3β and CDK5, miR-132 prevents hyperphosphorylation of tau, and so its downregulation observed in AD contributes to the formation of NFTs (Wang et al., 2023). Furthermore, miR-132 modulates genes associated with neuroinflammation and oxidative stress, detrimental processes in AD, because it normally downregulates the expression of pro-inflammatory cytokines like IL-1β and TNF-α which promotes a neuroprotective anti-inflammatory environment (Cui et al., 2022). Moreover, methyl-CpG binding protein 2 (MeCP2), a protein known to upregulate tau expression, is negatively regulated by miR-132. In essence, miR-132 acts as a molecular brake on MeCP2, preventing it from driving tau expression and thereby mitigating tau-mediated neurodegeneration (**Table 2**). Conversely, miR-132 deficiency observed in AD patients leads to increased MeCP2 levels, potentially creating a vicious cycle that exacerbates tau pathology (Xie et al., 2019; **Figure 2**). This extensive role of miR-132 in the pathogenesis of AD could mean it can be used as a biomarker to indicate AD or the risk of developing it by measuring its levels in blood or CSF. Additionally, by increasing miR-132 levels or targeting its downstream genes, AD progression may be slowed or prevented, though further research needs to be conducted on this possibility (Zhang and Bian, 2021).

### MicroRNA-146a and Alzheimer’s disease

MiR-146a is a brain-specific miR, particularly in the temporal lobe neocortex and hippocampus of AD mouse models (**Table 1**). Intriguingly, AD exhibits an upregulation of miR-146a in these very regions, making it emerge as a critical player in AD pathogenesis (Lukiw et al., 2008; **Figure 1**). Mechanistically, miR-146a exerts its influence through targeting genes critical for healthy neuronal communication. One such target is the rho-associated, coiled-coil containing protein kinase 1 (ROCK1) (Wang et al., 2016). miR-146a targets ROCK1, which plays a crucial role in phosphorylating PTEN at Ser380/Thr382/Thr383, resulting in the inactivation of the phosphatase activity of PTEN. When PTEN is phosphorylated (p-PTEN), it loses its ability to dephosphorylate tau, leading to tau hyperphosphorylation and the formation of NFTs. By inhibiting ROCK1, miR-146a reduces the phosphorylation of PTEN, thereby maintaining PTEN’s ability to dephosphorylate tau. In neural SH-SY5Y cells, overexpression of miR-146a significantly reduced ROCK1 protein translation and, as a result, decreased PTEN phosphorylation. This led to a decrease in tau hyperphosphorylation at sites such as Ser396. In contrast, silencing ROCK1 through siRNA reduced PTEN phosphorylation, mimicking the effects of miR-146a, and further supporting the role of ROCK1 in regulating PTEN activity and tau phosphorylation. In AD models, miR-146a upregulation coincides with reduced ROCK1 expression, which contributes to decreased p-PTEN levels and, consequently, an increased ability of PTEN to dephosphorylate tau. Thus, by reducing ROCK1 and subsequently, PTEN phosphorylation, miR-146a helps mitigate tau hyperphosphorylation and could potentially alleviate tau-related pathology in AD (Chen et al., 201). The potential to modulate miR-146a expression makes it a promising therapeutic target for AD. Researchers are exploring several strategies, including miR-146a inhibitors that specifically bind to miR-146a, preventing it from interacting with its target genes. This could potentially mitigate neuroinflammation to help slow AD progression (Liang et al., 2021).

Moreover, employing an Aβ_1–42_ treated mouse model of AD, researchers revealed a detrimental effect of miR-146a on cognitive function. Mice with increased miR-146a exhibited extended escape latency in the Morris Water Maze test, indicative of cognitive impairment. Mechanistically, the study demonstrates that miR-146a contributes to exacerbated Aβ deposition in the hippocampus, another key pathological feature of AD (Zhan-Kiang et al., 2023). To elucidate the underlying mechanism, the study explored the role of oxidative stress and the p38 Mitogen-activated protein kinase (MAPK) signaling pathway. The findings suggest that miR-146a promotes AD by triggering oxidative stress and neuroinflammation via activation of the p-p38 MAPK pathway (Liu et al., 2020). This proposes a mechanistic cascade where Aβ treatment triggers miR-146a upregulation, which in turn activates the p-p38 MAPK pathway. This activation leads to increased reactive oxygen species (ROS) production, ultimately promoting Aβ deposition in the hippocampus, and contributing to the cognitive decline and neuronal death characteristic of AD (Zhan-Kiang et al., 2023; **Figure 2**). From a therapeutic standpoint, this research offers promising insights. By targeting miR-146a or inhibiting the MAPK pathway, it might be possible to mitigate Aβ deposition, lessen oxidative stress, and potentially slow AD progression.

Importantly, human clinical studies also support miR-146a as a potential blood-based biomarker for AD. In a systematic review that analyzed peripheral blood profiling across 18 clinical studies—including more than 1000 AD patients and over 1200 healthy controls—miR-146a was consistently found to be significantly downregulated in AD patients compared to cognitively normal individuals in at least two independent cohorts (Wu et al., 2016). Although brain and CSF studies had previously reported miR-146a upregulation, these blood-based studies highlight potential compartment-specific expression dynamics, possibly reflecting compensatory or systemic inflammatory responses. Despite methodological differences across studies (e.g., plasma *versus* serum *versus* exosomes), miR-146a remained a consistently dysregulated miRNA in blood-based AD profiling. It was identified among a group of miRNAs that showed diagnostic sensitivity and specificity > 0.75 in distinguishing AD patients from controls, suggesting a practical and non-invasive diagnostic utility. Given its roles in neuroinflammation and tau phosphorylation, these findings highlight miR-146a as not only mechanistically relevant to AD pathology but also a strong candidate for peripheral biomarker development.

### MicroRNA-155 and Alzheimer’s disease

Neuroinflammation, a chronic and complex process involving glial cell activation and immune cell infiltration, is increasingly recognized as a critical contributor to AD pathogenesis (Moyse et al., 2022). T lymphocytes (T cells) are essential components of the adaptive immune system and play a multifaceted role in the CNS by providing neuroprotection through its role in producing neurotrophic factors (Walsh et al., 2014). Recent studies suggest that miR-155, a key regulator of immune responses, maybe a novel therapeutic target in AD by modulating T cell function. In the immune system, miR-155 is predominantly expressed in T cells and regulates their development, differentiation, activation, and effector functions (Zingale et al., 2021). miR-155 has been implicated in the regulation of various T cell subsets, including Th1, Th2, Th17, and regulatory T cells (Tregs). In physiological immune responses, miR-155 promotes the differentiation and effector functions of Th1 and Th17 cells, crucial for eliminating pathogens. Conversely, miR-155 deficiency impairs T cell responses and antigen presentation (Seddiki et al., 2014). Studies using AD mouse models have demonstrated increased miR-155 expression in the brain, particularly within microglia and astrocytes (**Figure 1**). Interestingly, this upregulation correlated with enhanced microglial activation and neuroinflammation (Guedes et al., 2014). Furthermore, in vitro studies suggest that miR-155 promotes the activation of pro-inflammatory Th1 and Th17 cells, potentially via targeting suppressor of cytokine signaling 1 (SOCS1). SOCS1 is a negative regulator of cytokine signaling, and its inhibition by miR-155 could lead to an amplified inflammatory response within the CNS, contributing to neuronal damage and disease progression (Song and Lee, 2015; **Figure 2**). The immunomodulatory properties of miR-155 suggest its potential as a therapeutic target for AD. By manipulating miR-155 levels or function, it may be possible to modulate T cell activity and dampen neuroinflammation (Gaudet et al., 2017; **Table 2**).

### MicroRNA-219 and Alzheimer’s disease

Encoded by genes located on Chromosome 17 and 2 in mouse genomes, miR-219 has been linked to multiple neurodegenerative diseases such as multiple sclerosis and leukodystrophies (Wang et al., 2017; **Table 1**). Recent investigations into miR-219 have unveiled it as a potential therapeutic against the neurodegenerative processes underlying AD. This miR exhibits brain-specific localization, with its expression levels concentrated within the entorhinal cortex, a region known for its early vulnerability in AD (Arnes et al., 2019; **Figure 1**). Intriguingly, postmortem brain tissue analysis revealed a significant downregulation of miR-219 in both AD and Tangle-predominant dementia (TPD) patients when compared to healthy controls. This regional and disease-specific downregulation suggests a potential link between miR-219 dysregulation and the neurodegenerative processes characteristic of AD (Santa-Maria et al., 2015). Mechanistically, miR-219 appears to exert its protective effects through direct targeting of the tau mRNA. By binding directly to the 3’-UTR of tau mRNA, miR-219 likely regulates its stability or translation efficiency, leading to a decrease in tau protein levels. This regulatory mechanism is further supported by the observed inverse correlation between miR-219 and tau protein levels (Arnes et al., 2019). Treatment with nerve growth factor (NGF), known to induce neurite outgrowth, caused a transient decrease in miR-219 levels followed by myelin degeneration. When miR-219 was expressed in NGF-treated cells, there was a significantly increased oligodendrogenesis accompanied by the inhibition of neurite outgrowth, all hallmarks of AD (Brandi et al., 2021).

Further strengthening this link is the coordinated regulation of tau kinases by miR-219. *In silico* analysis identified three predicted miR-219 targets: Calcium/calmodulin-dependent protein kinase 2 gamma subunit (CAMK2γ), Tau tubulin kinase 1 (TTBK1), and GSK3Fβ, all of which are implicated in the generation of abnormal hyperphosphorylated tau. Notably, the protein levels of these three kinases were found to be upregulated in the entorhinal cortex of AD brains, exhibiting an inverse correlation with the downregulated miR-219 levels. Moreover, miR-219 directly interacts with the 3’-UTR of these kinase mRNAs, potentially influencing their translation and ultimately leading to a decrease in tau phosphorylation. This coordinated regulation of tau and its kinases by miR-219 presents a unifying mechanism by which it might counteract the detrimental effects of tau pathology in AD (Li et al., 2019). The functional significance of miR-219 in AD pathogenesis was further established in vivo models employing fruit flies expressing human tau. Overexpression of miR-219 in these models resulted in a partial reversal of the detrimental effects caused by tau, while conversely, inhibiting miR-219 exacerbated these effects. Similar observations were made in human cell cultures, where miR-219 mimics significantly reduced tau expression by directly interacting with the tau 3’-UTR. Additionally, miR-219 deficiency in vivo led to aggravated tau pathology, neurodegeneration, and impaired learning and memory in these models (Santa-Maria et al., 2015; **Figure 2**). Importantly, a human cohort study using CSF samples from 22 AD patients and 28 disease controls revealed that miR-219 is significantly downregulated in AD. This study applied Openarray RT-qPCR to analyze over 1,100 unique miRNAs and identified miR-219 among nine “informative” biomarkers using the measure of relevance statistical method. Multivariate analysis confirmed the differential expression of miR-219 between AD and control groups, with Bonferroni-corrected statistical significance. Furthermore, Ingenuity pathway analysis predicted miR-219 to target the MAPT gene (which encodes tau), further reinforcing its mechanistic role in AD pathology (Denk et al., 2015; **Table 2**). These findings collectively support the notion that miR-219 dysregulation is central to AD pathogenesis and highlight its potential as a therapeutic target for the treatment or prevention of AD and related tauopathies.

### MicroRNA-501-3p and Alzheimer’s disease

MiR-501-3p is an established brain-specific miR linked to various neurological disorders including schizophrenia and dementia (Devara et al., 2023; **Table 1**). A previous study has explored the potential of miR-501-3p, another brain-specific microRNA, as a biomarker for AD progression (Siedlecki-Wullich et al., 2021). Researchers measured miR-501-3p levels in both the blood serum and brain tissues collected from AD patients and healthy controls, showing an inverse relationship between serum and brain levels. Serum miR-501-3p levels were significantly lower in AD patients compared to healthy individuals. Conversely, brain tissue from AD patients, particularly in regions severely affected by the disease, showed a remarkable upregulation of miR-501-3p in the temporal cortex. Furthermore, both serum and brain levels of miR-501-3p correlated with the Braak NFT stages, a measure of disease severity in AD (Hara et al., 2017). Hsa-miR-501-3p overexpression in cultured cells suggests that the upregulation of miR-501-3p in the brain induced the downregulation of 128 genes involved in DNA replication and mitotic cell cycle (Hara et al., 2017). This finding is intriguing because inappropriate cell cycle reentry in postmitotic neurons, which leads to apoptotic cell death, is an indication of senile plaque (SP) and NFTs in AD brains. Alternatively, other research points to a potential role for miR-501-3p in regulating synaptic plasticity, which is essential for learning and memory, by targeting the glutamate ionotropic receptor AMPA type subunit 1 (Gria1) gene and therefore mediating the expression of α-amino-3-hydroxy-5-methyl-4-isoxazolepropionic acid (AMPA) receptor subunit glutamate receptor 1 (GluA1) in dendrites (**Figure 2**). Since synaptic dysfunction is a well-established characteristic of AD, this pathway might also be relevant in the disease process and could potentially serve as a biomarker reflecting disease progression (Kumar et al., 2022; **Table 2**).

Recent findings further extend the role of miR-501-3p by identifying its interaction with *GABRA1*, the gene encoding the alpha1 subunit of the GABA-A receptor, indicating that it may regulate both excitatory and inhibitory neurotransmission (Kumar et al., 2022). This dual targeting suggests that miR-501-3p could contribute to synaptic imbalance in AD, disrupting neural circuit function. Additionally, the biological basis for the inverse brain-serum distribution pattern of miR-501-3p remains incompletely understood. It is hypothesized that blood-brain barrier dysfunction, altered exosomal sorting, or intracellular retention mechanisms may underlie this phenomenon, compounded by potential differences in miRNA degradation rates across compartments. From a therapeutic perspective, miR-501-3p presents challenges due to the limited ability of miRNA-targeted therapies to cross the blood-brain barrier. Though approaches involving lipid nanoparticles, viral vectors, or exosome-based systems are in development, they require further refinement to achieve brain-region specificity and safety. Moreover, given miR-501-3p’s broad interactome, off-target effects remain a significant concern, underscoring the need for high-throughput mapping techniques such as cross-linking and immunoprecipitation followed by sequencing, argonaute immunoprecipitation, and single-cell transcriptomics to delineate its full range of gene targets.

There are also critical gaps regarding its cell-type-specific expression and temporal dynamics throughout AD progression. Whether miR-501-3p dysregulation occurs predominantly in neurons, astrocytes, or microglia—and whether it drives or reflects disease pathology—remains to be clarified. Future research should therefore focus on resolving these spatial and temporal aspects using spatial transcriptomics, longitudinal studies, and human-relevant AD models. Advances in cell-type-targeted delivery strategies, such as neuron-specific viral vectors or ligand-conjugated nanoparticles, will also be key to translating miR-501-3p-based interventions from bench to bedside. Ultimately, miR-501-3p stands out as a multifunctional regulator in AD, with mechanistic involvement in cell cycle reentry, synaptic disruption, and potential diagnostic utility. Its complexity demands precision in both mechanistic understanding and therapeutic application, but also offers a promising avenue for targeted intervention in AD.

### MicroRNA-502-3p and Alzheimer’s disease

Belonging to the same family and seed sequence as miR-501-3p, miR-502-3p has been associated with a wide variety of diseases, including type 2 diabetes, osteoporosis, and dementia. It stands out for its brain-enriched expression, suggesting a critical role in neuronal function and dysfunction, particularly in neurodegenerative diseases such as AD. Recent research highlights a compelling link between miR-502-3p and synaptic dysfunction, a hallmark of AD (Meftah and Gan, 2023). A previous study has shown that miR-502-3p is significantly elevated in synaptosomes—specialized structures essential for neuronal communication—in AD patients compared to healthy individuals (Sharma et al., 2024; **Figure 1**). This elevation correlates with disease progression and implicates miR-502-3p in the underlying pathophysiology of AD (Xu et al., 2020). Functionally, miR-502-3p targets the gamma-aminobutyric acid type A receptor subunit alpha1 (GABRA1) gene, critical for GABAergic synaptic inhibition (Kumar et al., 2022). GABAergic dysfunction contributes to the excitatory/inhibitory imbalance observed in AD (Rivera et al., 2023). Both miR-502-3p and miR-501-3p negatively regulate GABRA1, and high levels of miR-502-3p have been shown to reduce GABRA1 protein levels and GABA-induced electrical currents in neurons (**Figure 2**). Conversely, inhibition of miR-502-3p increases GABRA1 expression and restores GABAergic function, suggesting its potential as a therapeutic target in AD (Kumar et al., 2022; **Table 2**).

What makes miR-502-3p particularly unique is its compartmental precision and functional specificity. Unlike miR-501-3p, which affects broader pathways like the cell cycle and synaptic regulation, miR-502-3p appears to act primarily at the synapse—specifically within inhibitory circuits. This synaptic enrichment offers an opportunity for ultra-targeted therapeutic modulation, potentially minimizing systemic off-target effects. Moreover, the functional convergence between miR-502-3p and miR-501-3p on GABRA1 regulation underscores the possibility that miRNA families operate cooperatively or redundantly to maintain synaptic balance. This layered control might ensure homeostatic stability under normal conditions but also increase vulnerability when miRNA expression is disrupted, as in AD. Despite this promise, several translational challenges remain. Chief among them is the difficulty of delivering miR-502-3p-targeted therapies across the BBB. AntagomiRs and antisense oligonucleotides (ASOs), though effective *in vitro*, often struggle to reach the CNS in sufficient concentrations. While intracerebral delivery has shown success in preclinical models, it is invasive and unsuitable for long-term use in human patients. Emerging delivery strategies—such as exosome-based vectors, synapse-targeting nanoparticles, and neuron-specific viral constructs—are under investigation but are still in developmental stages. Another major limitation is the incomplete mapping of miR-502-3p’s interactome. While GABRA1 is a validated target, miRNAs typically regulate multiple transcripts. Without a full understanding of its synaptic or glial targets, therapeutic modulation could carry the risk of off-target effects. Furthermore, the mechanism by which miR-502-3p localizes specifically to synapses remains unclear—whether it involves activity-dependent trafficking, RNA-binding proteins, or vesicular transport systems is still under investigation.

To realize the therapeutic potential of miR-502-3p, several research avenues are essential. High-resolution interactome mapping using cross-linking and immunoprecipitation followed by sequencing, miRNA pull-down assays, and mass spectrometry will be necessary to clarify target networks. Spatially resolved transcriptomics and proteomics in AD models and postmortem tissues will further define its temporal and regional expression patterns. Parallel development of delivery technologies that can selectively target inhibitory neurons or synaptic vesicle compartments will be crucial for clinical translation. In addition to its therapeutic relevance, miR-502-3p holds strong potential as a biomarker for early-stage synaptic dysfunction. Its synaptic enrichment and correlation with disease severity position it as a minimally invasive marker accessible through EVs in CSF or plasma. Platforms that isolate synaptic vesicle-derived EVs could allow real-time monitoring of synaptic integrity and therapeutic responses. Overall, while miR-502-3p’s unique localization and narrow targeting profile make it a compelling candidate for both biomarker development and precision therapeutics in AD, further investigation is required to address key translational challenges. Future research should prioritize delivery strategies, target validation, and longitudinal biomarker studies to unlock its full potential in combating synaptic pathology in AD.

## Collective Impact and Pathway-Centric Targets of Brain-Specific MicroRNAs in Alzheimer’s Disease

Recent findings have provided valuable insights that rather than acting in isolation, many miRNAs function within overlapping regulatory networks that target key signaling hubs in amyloid processing, tau phosphorylation, neuroinflammation, synaptic plasticity, mitochondrial dynamics, and autophagy (Li et al., 2024). In the context of APP processing and Aβ production, miR-107 plays a central role by targeting *BACE1*, thereby limiting the cleavage of APP and subsequent Aβ generation. Its early downregulation in AD brains correlates with increased *BACE1* levels and Aβ plaque burden. miR-124 similarly suppresses *BACE1* and maintains synaptic homeostasis through the regulation of calcium signaling proteins such as *RyR3*, positioning it as a dual-function regulator of amyloid metabolism and synaptic integrity. Importantly, miR-124 and miR-107 act synergistically, and their concurrent reduction disrupts multiple layers of neuroprotection. Furthermore, miR-128 contributes to amyloid regulation by inhibiting *APPBP2*, a cofactor in APP processing, and enhancing autophagic clearance through repression of mTOR signaling. These three miRNAs—miR-107, miR-124, and miR-128—form a tightly linked axis that connects amyloidogenesis to metabolic and synaptic homeostasis.

Tau pathology, the second major neuropathological hallmark of AD, is modulated by miR-132, miR-125b, miR-219, and miR-26b. Among them, miR-132 is one of the most consistently downregulated miRNAs in AD brains. It directly suppresses *GSK3*β and *CDK5*, major tau kinases, and supports *CREB* signaling, essential for memory and neuronal survival. In contrast, miR-125b is upregulated in AD and promotes tau hyperphosphorylation by inhibiting phosphatases such as *DUSP6* and pro-survival proteins such as *Bcl-W*. miR-219, a less frequently studied but crucial miRNA, targets multiple tau regulators including *CAMK2γ*, *TTBK1*, and *GSK3*β, thus acting as a broad-spectrum modulator of tau dynamics. miR-26b, which is aberrantly expressed in AD, also targets *GSK3*β and influences cell cycle re-entry in neurons—a deleterious process associated with tau accumulation and neuronal death. Together, this group of miRNAs governs tau homeostasis by fine-tuning both kinase and phosphatase activity, and their dysregulation results in a convergence toward increased tau aggregation and toxicity. Neuroinflammation, increasingly recognized as a key driver of AD progression, is heavily influenced by miR-146a, miR-155, and miR-125b. miR-146a targets *IRAK1* and *TRAF6*, components of the TLR/NF-κB pathway, and acts as a negative regulator of excessive inflammation. However, in chronic AD pathology, sustained upregulation of miR-146a may reflect an exhausted or maladaptive response, failing to resolve microglial overactivation. miR-155, conversely, is a pro-inflammatory miRNA that amplifies cytokine production, particularly *TNF-α* and *IL-6*, by targeting negative regulators of immune signaling such as *SOCS1*. miR-125b’s dual role extends into inflammation, where it suppresses *PTEN* and *ROCK1*, fostering microglial reactivity and neurotoxicity. Notably, all three miRNAs are enriched in glial cells surrounding amyloid plaques, reinforcing their contribution to the non-neuronal inflammatory milieu in AD (**Table 2**).

The maintenance of synaptic integrity and plasticity is another domain where these miRNAs exert coordinated control. miR-124 and miR-132, both downregulated in AD, play indispensable roles in sustaining dendritic spine formation, neurotransmitter receptor trafficking, and CREB-mediated synaptic signaling. Their loss precedes cognitive impairment and is closely linked to hippocampal atrophy. miR-128 and miR-9 add further regulatory depth—miR-128 supports synaptic resilience by promoting glutamatergic receptor insertion, while miR-9 regulates neuronal differentiation and axonal extension. Of particular interest, miR-9 displays dynamic expression, increasing in early AD stages and declining later. This biphasic pattern may reflect a compensatory response that ultimately fails under sustained pathology. miR-26b, although less studied in this context, also influences synaptic health by targeting cytoskeletal and cell cycle regulators. Emerging players in this regulatory network are miR-501-3p and miR-502-3p, which have been detected in both CSF and serum of AD patients. Their expression levels correlate with Braak staging and cognitive severity, suggesting value as non-invasive biomarkers. However, the functional characterization of their direct targets remains limited. Preliminary data implicate roles in inflammatory modulation and neuronal stress responses, but detailed mechanistic studies are lacking—a key knowledge gap in the current literature. Moreover, the spatiotemporal regulation of miR-501-3p and miR-502-3p remains unclear, warranting longitudinal studies to assess their stage-specific roles and responsiveness to therapeutic interventions.

Across these functional domains, miRNAs act as master regulators of entire signaling modules rather than single-gene silencers. The co-regulation of shared targets—such as *GSK3*β, *BACE1*, and *NF-κB* pathway components—by multiple miRNAs (e.g., miR-132, miR-125b, miR-124, and miR-146a) reflects an intricate layer of redundancy and feedback that stabilizes brain homeostasis under physiological conditions. In AD, the collapse of this miRNA network due to either overexpression or depletion of key miRs leads to the simultaneous dysregulation of multiple pathogenic cascades (**Figure 2**). Moreover, temporal and regional shifts in miRNA expression provide diagnostic and therapeutic insight. For example, miR-132 and miR-124 are consistently reduced in the hippocampus and entorhinal cortex—regions affected early in AD. Conversely, miR-125b and miR-155 are elevated in the temporal cortex and amygdala, correlating with advanced neuroinflammation. These spatial patterns suggest that miRNAs may not only reflect disease stage but also local vulnerability, making them promising candidates for precision neuromodulation (**Figure 1**). As therapeutic strategies evolve, the integration of miR-based approaches with conventional AD therapies holds promise. For instance, combining anti-miR strategies to suppress pro-inflammatory miRs with miR mimics that enhance synaptic resilience could produce synergistic effects. The emerging field of combinatorial miR modulation—using cocktails tailored to an individual’s miR signature—represents a leap toward precision neuromedicine. Delivery technologies such as exosome-based vectors and adeno-associated virus systems further enhance the feasibility of targeted brain-specific miR therapy (Walgrave et al., 2021).

## Biological Relevance of Brian-Specific MicroRNAs in Alzheimer’s Disease

As seen, brain-specific miR is incredibly powerful in its therapeutic and biomarker potential in AD due to its unique location in the CNS and dysregulation based on the disease’s progression. Some miRs, such as miR-124, are naturally abundant in the brain and promote neuron health and function through its regulation of specific genes, such as RyR3 (Liu et al., 2022; **Table 2**). However, in AD, these miRs are often downregulated, so researchers are developing methods to increase its expression. This could involve introducing synthetic copies of these miRs or manipulating the cellular machinery responsible for miR production. By promoting the down-regulated miR, scientists hope to enhance the ability of the brain to clear harmful protein deposits like Aβ plaques. Conversely, some miRs become abnormally upregulated in AD and contribute to the disease process. An example is miR-146a, which has been linked to tau phosphorylation due to its upregulation inhibiting ROCK1 protein translation (Wang et al., 2016; **Figure 2**). Therapeutic strategies aim to silence or inhibit such upregulated miR. This could involve using antisense oligonucleotide therapies, essentially molecules designed to bind specifically to the miR and block its function, preventing it from contributing to AD pathogenesis. Additionally, by analyzing these changes in miR in CSF of patients, a minimally invasive procedure, researchers can detect AD earlier than conventional methods. This could hold significant clinical value, allowing for intervention before the onset of severe symptoms. Furthermore, blood analysis offers a potentially even less invasive approach for miR-based AD biomarkers. Tracking longitudinal changes in miR profiles within individual patients might also provide valuable insights into disease progression by guiding treatment decisions and potentially personalizing therapeutic strategies.

## Therapeutic Potential of Brain-Specific MicroRNAs in Alzheimer’s Disease

miR-26b holds possible therapeutic potential because it is overexpressed specifically in the temporal cortex and the miR disrupts critical checkpoints in the neuronal cell cycle, leading to neuronal death (Zhang et al., 2020). Developing anti-miR-26b molecules, which are delivered regionally using nanoparticles, could specifically target the upregulated miR-26b in the temporal cortex (**Figure 1**). This targeted approach could promote neuronal survival in vulnerable brain regions while minimizing side effects in other areas (Absalon et al., 2013). Compared to anti-Aβ therapies such as lecanemab or donanemab, which target extracellular amyloid with limited regional specificity, miR-26b inhibition offers intracellular modulation tailored to local cortical dysregulation. This regional and intracellular precision represents a potential advantage in addressing early, localized neurodegeneration.

The inverse correlation observed in individuals with lower miR-107 in the cerebral cortex and higher levels of BACE1 mRNA, especially in advanced AD pathology, suggests that miR-107 could serve as a valuable therapeutic. Research has also shown that miR-107 directly regulates BACE1 by targeting its mRNA, thereby controlling its translation into protein (Zhao et al., 2007; **Figure 2**). By increasing miR-107 levels, researchers hope to suppress BACE1 production and consequently reduce Aβ plaque formation, a critical pathological hallmark of AD. The neuronal specificity of miR-107 within the cerebral cortex makes it an ideal candidate for therapeutic strategies aimed at directly regulating BACE1 in these critical brain areas. In contrast to monoclonal antibodies that clear existing Aβ, miR-107 offers a preventative approach by downregulating the Aβ-producing enzyme upstream of plaque formation. This allows for potential intervention earlier in the disease course and may avoid the inflammatory responses observed with passive immunotherapy.

MiR-124 exhibits neuroprotective properties because it has an inverse relationship with BACE1, the enzyme responsible for generating Aβ, a major component of amyloid plaques. Reduced miR-124 leads to increased BACE1 levels, potentially accelerating Aβ production. Conversely, overexpressing miR-124 appears to mitigate Aβ-induced neuronal toxicity. This suggests that increasing miR-124 levels could offer neuroprotection by regulating BACE1 activity and preventing Aβ-mediated damage (Fang et al., 2012). miR-124 can also regulate PTPN1, a protein involved in memory function. When PTPN1 is downregulated due to low miR-124, it contributes to synaptic abnormalities and memory decline (Kumar and Reddy, 2020). By increasing miR-124 levels and regulating PTPN1, neuronal protection can be provided and cognitive function will most likely improve in AD patients. This broad targeting strategy may provide greater functional rescue than current small-molecule BACE1 inhibitors, which have shown limited efficacy and adverse cognitive effects in clinical trials.

Despite the complexities surrounding its expression, miR-128 exhibits several well-defined neuroprotective properties for therapeutic potential by regulating Aβ production by suppressing NF-κB and APPBP2 (Jin et al., 2018). Additionally, it enhances autophagy, a cellular process responsible for clearing Aβ plaques, by targeting mTOR and making it a promising agent for therapeutics in AD (Decressac et al., 2013). Future research will explore strategies to both enhance the neuroprotective effects of miR-128 and mitigate any unintended consequences. This could involve targeted delivery of miR-128 to specific brain regions or utilizing gene editing techniques for precise expression regulation. Additionally, researchers may aim to prevent PPARγ downregulation by targeting the pathways involved in its interaction with miR-128. Unlike tau-targeting antibodies such as gosuranemab or semorinemab, which largely neutralize extracellular tau, miR-128 modulates intracellular tau phosphorylation indirectly via kinase regulation, potentially offering broader effects on tau-mediated neurodegeneration.

MiR-219 emerges as a promising therapeutic candidate for AD due to its unique properties that enable targeted intervention and regulation of core pathological processes. Unlike traditional medications with widespread effects, miR-219 exhibits high expression specifically within the entorhinal cortex, a brain region particularly vulnerable in early AD stages. This regional specificity allows for a pinpointed therapeutic approach, directly addressing the most affected areas at the onset of the disease (Arnes et al., 2019). Furthermore, the significant downregulation of miR-219 observed in AD patients suggests a critical role in healthy brain function. Restoring or supplementing these depleted miR-219 levels could potentially reverse some of the detrimental effects caused by AD. Mechanistically, miR-219 directly targets the mRNA of tau, potentially decreasing its overall production. Additionally, it takes aim at the mRNAs of tau kinases, enzymes responsible for the abnormal hyperphosphorylation of tau. By simultaneously regulating both tau and its kinases, miR-219 offers a unified therapeutic approach that could be more effective than therapies focusing on just one aspect of tau pathology. In fact, studies using fruit flies and human cell cultures provide compelling preclinical evidence for the therapeutic potential of miR-219, demonstrating its ability to reverse detrimental effects caused by tau, reduce tau expression, and mitigate tau pathology and neurodegeneration (Santa-Maria et al., 2015). Compared to single-target therapies, the ability of miR-219 to downregulate both tau and its kinases may yield a more durable impact on tau pathology progression.

Additionally, the therapeutic potential of miR-501-3p lies in its ability to regulate genes involved in DNA replication and the mitotic cell cycle, processes typically inactive in mature neurons. This finding is particularly relevant in AD, where inappropriate cell cycle re-entry in postmitotic neurons contributes to the formation of NFTs. By regulating this process, miR-501-3p could potentially mitigate neurodegeneration (Hara et al., 2017). Additionally, research suggests a role for miR-501-3p in regulating synaptic plasticity, crucial for learning and memory, by targeting the GluA1 receptor subunit in dendrites (Hu et al., 2015). Since synaptic dysfunction is another well-established characteristic of AD, this pathway offers another potential therapeutic target. By potentially regulating GluA1 expression and influencing synaptic function, miR-501-3p could contribute to mitigating cognitive decline in AD. Its unique inverse expression profile, upregulated in brain tissue but downregulated in peripheral blood, enhances its value as both a mechanistic regulator and a diagnostic biomarker. This contrasts with amyloid PET imaging, which, while useful, is costly, less accessible, and does not inform about therapeutic responsiveness.

Similar to miR-501-3p, miR-502-3p and its high levels in the synaptosomes correlate with decreased GABRA1 protein by regulating GABRA1 expression, potentially leading to reduced GABAergic signaling and contributing to the very synaptic problems and impaired communication between neurons observed in AD (Sharma et al., 2024). Conversely, research suggests that inhibiting miR-502-3p increases GABRA1 protein and GABA currents, potentially restoring normal function. Since malfunctioning GABA neurons contribute to AD, regulating GABA through miR-502-3p inhibition emerges as a promising therapeutic strategy by potentially restoring proper neuronal communication in AD patients (Sharma et al., 2024). This strategy may offer more targeted synaptic modulation than systemic GABA agonists, which often induce sedation or tolerance. By focusing on the underlying post-transcriptional regulation, miR-502-3p inhibition could normalize inhibitory signaling in a regionally and molecularly selective manner. Despite their promise, miRNA-based therapies face significant translational challenges. A primary barrier is efficient delivery across the BBB, which restricts the entry of most systemically administered RNA therapeutics. Intrathecal or intracerebral administration, while effective, is invasive and not ideal for chronic conditions like AD (**Table 2**). To overcome this, emerging strategies include the use of lipid nanoparticles, exosomes engineered for neuronal targeting, and viral vectors (e.g., AAVs) with CNS tropism. However, these methods vary in safety, immunogenicity, scalability, and cell-type specificity.

Another major concern is off-target effects. Unlike antisense oligonucleotides or monoclonal antibodies with high specificity, miRNAs regulate gene expression through partial base pairing, allowing them to bind to hundreds of potential mRNA targets. This increases the risk of unintended gene silencing, especially in non-target tissues. Cell-type-specific delivery systems and next-generation chemical modifications (e.g., locked nucleic acids, phosphorothioate backbones) are being explored to mitigate this risk. Furthermore, miRNA stability, biodistribution, and degradation in circulation remain hurdles. Endogenous miRNAs are often protected in exosomes or protein complexes, but exogenous miRNA mimics or inhibitors must be engineered to resist nucleases while preserving biological activity. Robust pharmacokinetic and pharmacodynamic modeling is essential to ensure consistent dosing and minimize toxicity.

## Biomarker Potential of Brain-Specific MicroRNAs in Alzheimer’s Disease

MiR-501-3p has gained recognition as a compelling biomarker candidate in AD due to its distinct and inverse expression profile between the peripheral circulation and the brain. In AD patients, serum levels of miR-501-3p are significantly reduced, while in contrast, brain tissue, particularly in regions with advanced pathology such as the temporal cortex, exhibits marked upregulation (Hara et al., 2017). This inverse serum-brain correlation is especially intriguing because both compartments’ miR-501-3p levels are tightly correlated with Braak NFT stages, a validated histopathological measure of AD severity. This suggests that as the disease progresses and NFT burden increases, miR-501-3p becomes increasingly sequestered or upregulated in the brain, potentially reflecting its functional involvement in disease mechanisms and neuronal stress responses. Mechanistically, this inverse pattern may be attributed to pathology-driven redistribution, whereby miR-501-3p is preferentially retained or overexpressed in degenerating brain regions, possibly as a failed compensatory mechanism or due to impaired transport mechanisms across the BBB. Alternatively, altered exosomal packaging, degradation in serum, or impaired efflux from the CNS could contribute to the reduced peripheral levels. This compartment-specific regulation enhances the potential of miR-501-3p as a dual-compartment biomarker: low serum levels could serve as a minimally invasive screening tool, while elevated brain levels could reflect disease burden or target engagement in therapeutic contexts.

Similarly, miR-502-3p has emerged as a promising AD biomarker due to its synapse-specific enrichment and tight correlation with both neuropathological and biochemical hallmarks of the disease. Unlike many miRNAs that exhibit diffuse expression changes in bulk tissue, miR-502-3p has been shown to be specifically enriched within synaptosomes, isolated synaptic terminals, of AD brains (Meftah and Gan, 2023; Sharma et al., 2024). This compartmentalized and functionally relevant expression profile offers a highly specific signal for detecting synaptic degeneration, which is one of the earliest pathophysiological events in AD. Its presence in CSF exosomes, tiny vesicles shed by neurons and synapses, also means that miR-502-3p can be measured through minimally invasive liquid biopsies, making it a powerful tool for early detection, staging, and monitoring of therapeutic responses. Together, miR-501-3p and miR-502-3p offer complementary diagnostic value. miR-501-3p tracks neurofibrillary pathology via a periphery-to-CNS shift, while miR-502-3p reflects synaptic pathology at the molecular level, accessible via CSF or potentially blood-derived exosomes. This dual-marker strategy could enable more granular stratification of disease stages and allow for dynamic monitoring of AD progression or response to disease-modifying therapies (**Table 2**).

However, translating these findings into clinical application, whether as biomarkers or therapeutic targets, requires overcoming significant barriers, particularly those related to miRNA delivery and specificity. Chief among these is the challenge of crossing the BBB. Systemically administered miRNA mimics, antagomiRs, or inhibitors typically exhibit limited CNS penetration, necessitating high doses or invasive delivery methods. Current strategies, such as lipid nanoparticles, viral vectors, and exosome-based delivery systems, are under active investigation, but each comes with limitations. Nanoparticles may accumulate non-specifically in peripheral tissues, while viral vectors pose risks of immune activation and long-term genomic integration. An equally critical challenge is targeting specificity. miRNAs function via partial sequence complementarity, allowing them to bind to multiple mRNA targets, sometimes hundreds, raising the risk of off-target effects. In the case of miR-501-3p and miR-502-3p, while key targets such as GABRA1 (gamma-aminobutyric acid receptor subunit alpha1) and GRIA1 (glutamate receptor AMPA subunit) have been validated, their full interactomes remain incompletely characterized. Unintended repression of unrelated but essential genes could lead to adverse outcomes, especially in non-neuronal tissues. This underscores the need for high-throughput target validation (e.g., cross-linking and immunoprecipitation followed by sequencing and argonaute-RNA immunoprecipitation), and the development of context- and cell-specific delivery systems to confine therapeutic effects to the desired neuronal subtypes or brain regions. Moreover, variability in miRNA stability, circulation half-life, and EV incorporation can complicate assay development for biomarker purposes. Standardized protocols for sample collection, miRNA extraction, normalization, and interpretation are critical before miR-501-3p and miR-502-3p can be confidently used in clinical diagnostics.

## Future Directions

Brain-specific miRs have emerged as a promising avenue for both therapeutic intervention and diagnostic breakthroughs in AD. Unlike other miRs, these brain-enriched regulators wield precise control over neuronal function and health. Their dysregulation in AD offers a unique window into molecular underpinnings of the disease. To fully exploit their potential, future research should prioritize deciphering the intricate regulatory landscape of brain-specific miR-mRNA interactions within the neuronal landscape. Advanced techniques such as RNA capture sequencing coupled with deep sequencing will illuminate these critical events, exposing potential therapeutic targets. The brain-specific nature of these miRs presents a distinct advantage for targeted therapeutic development. By leveraging delivery systems with precise neuronal tropism, such as viral vectors with specific neuronal targeting or engineered nanoparticles adorned with brain-targeting ligands, researchers can ensure localized action within the brain, minimizing off-target effects in peripheral tissues. Furthermore, brain-specific miRs regulating BBB permeability hold immense potential for enhancing delivery of established AD therapeutics. This strategy essentially unlocks the fortress of the brain, allowing for a multi-pronged therapeutic attack.

Brain-specific miR signatures in biofluids such as blood or CSF offer a revolutionary approach for AD diagnosis and monitoring. Large-scale studies employing targeted miR panels can identify disease-specific signatures for early detection and disease progression. Tracking changes in these miR profiles over time could serve as a real-time window into treatment response, allowing for personalized treatment adjustments. Unraveling the influence of lifestyle factors such as diet, exercise, and stress on brain-specific miR expression is also crucial. Studies investigating the impact of dietary components or exercise regimens on miR profiles in AD models could pave the way for lifestyle interventions aimed at optimizing brain-specific miR expression for AD prevention or management. Dietary modifications or personalized exercise regimens could be tailored to optimize brain-specific miR expression, potentially delaying or even preventing AD onset one day with further studies.

As brain-specific miR-based therapies progress, careful consideration of ethical implications is paramount. Rigorous preclinical studies are essential to assess potential off-target effects and long-term safety of manipulating brain-specific miR expression. Additionally, informed consent processes for clinical trials need to be meticulously designed to address the unique complexities of targeting the brain and the potential cognitive decline associated with AD. Overall brain-specific miRs’ exquisite control over neuronal function and their unique disease signatures position them as the cornerstone of future AD therapies and diagnostics. By harnessing the power of brain specificity, researchers can unlock a new era of personalized medicine, offering hope for improved patient outcomes and a future free from the devastating effects of AD.

## Conclusions

This investigation into the role of brain-specific miRs in AD unveils a complex regulatory network with profound implications for disease pathogenesis and therapeutic development. These findings demonstrate a stage-dependent dysregulation of numerous miRs, impacting a diverse array of cellular processes critical for neuronal health. The observed alterations in autophagy, cell cycle control, tau phosphorylation, Aβ production, and neuroinflammation highlight the multifaceted influence of miRs on AD progression. These findings strongly suggest that targeting specific brain-specific miRs holds immense therapeutic potential. Upregulation of neuroprotective miRs, such as miR-132, which has been shown to promote neuronal survival and neurite outgrowth, could offer a novel strategy to counteract neurodegeneration. Conversely, downregulating detrimental miRs, such as miR-125b, known to exacerbate Aβ plaque formation and neuroinflammation, could be a promising therapeutic avenue.

Furthermore, the potential of brain-specific miRs as diagnostic and prognostic biomarkers for AD is highly attractive. Their accessibility in peripheral biofluids, such as CSF or blood, offers a minimally invasive approach for early disease detection and monitoring treatment efficacy. Studies have shown altered miR profiles in AD patients compared to controls, suggesting their potential for diagnosis. Additionally, the stage-dependent nature of miR dysregulation may enable the identification of patients at different stages of AD, facilitating the development of targeted interventions. However, significant challenges remain before miR-based therapies can be translated into clinical practice. A thorough understanding of the intricate network of miR-mRNA interactions and their influence on downstream signaling pathways in the context of AD is crucial. Additionally, developing efficient and safe delivery methods for miR therapeutics specifically to the brain remains a hurdle. Strategies such as nanoparticle-based delivery systems or viral vectors hold promise, but further research is necessary to ensure their efficacy and safety. Despite these challenges, the exploration of brain-specific miRs in AD research presents a groundbreaking opportunity for the development of novel diagnostics and therapeutics. Further research focused on deciphering miR-mRNA networks, developing targeted delivery systems, and conducting robust clinical trials is warranted to translate this exciting area of research into tangible benefits for patients suffering from AD.

## Data Availability

*Not applicable*.
